# Spindle Power Is Not Affected after Spontaneous K-Complexes during Human NREM Sleep

**DOI:** 10.1371/journal.pone.0054343

**Published:** 2013-01-10

**Authors:** Andreas M. Koupparis, Vasileios Kokkinos, George K. Kostopoulos

**Affiliations:** Neurophysiology Unit, Department of Physiology, Medical School, University of Patras, Rion, Greece; University of California, Riverside, United States of America

## Abstract

K-complexes and sleep spindles often grouped together characterize the second stage of NREM sleep and interest has been raised on a possible interaction of their underlying mechanisms. The reported inhibition of spindles power for about 15 seconds following evoked K-complexes has implications on their role in arousal. Our objective was to assess this inhibition following spontaneous K-complexes. We used time-frequency analysis of spontaneous K-complexes selected from whole-night EEG recordings of normal subjects. Our results show that spindles are most often observed at the positive phase following the peak of a spontaneous KC (70%). At latencies of 1–3 s following the peak of the K-complex, spindles almost disappear. Compared to long-term effects described for evoked KCs, sleep spindle power is not affected by spontaneous KCs for latencies of 5–15 s. Observation of the recurrence rate of sporadic spindles suggests that the reduction of power at 1–3 s most likely reflects a refractory period of spindles lasting for 1–2 s, rather than an effect of KCs. These results suggest that the mechanisms underlying spontaneous KCs do not affect spindle power as in the case of evoked KCs.

## Introduction

The sleep spindle and the K-complex (KC) are the electroencephalographic (EEG) hallmarks of the second stage of human non-rapid eye movement (NREM) sleep. Defined as a high-voltage biphasic slow wave with a negative phase that may be followed by a positive phase, the KC is one of the most distinguished graphoelements of the EEG [1], [2]. The sleep spindle, an oscillatory rhythm (11–15 Hz) of a waxing and waning shape, lasting 0.5–2 s is also a clearly distinguishable EEG event unique to sleep [3]. Fast (∼13–15 Hz) and slow (∼11–12 Hz) spindles are readily distinguishable with maximal power in centro-parietal and centro-frontal regions respectively [4]–[6].

A notable observation since the first description of the KC [7] is that it may appear either spontaneously or after a sensory stimulus, in which case it is named 'evoked'. This fact has led to a series of experiments over the decades on a search of its functional significance, with many researchers correlating the appearance of a KC with autonomic alterations and forthcoming arousals [8]–[12], thus assuming it is an arousing reaction. On the other hand, some suggest that the KC represents a sleep-protecting mechanism averting arousals [2]. Finally, a combined view of KC being a sleep promoting reaction to arousing stimuli seems to gain acceptance [13]. The role of the sleep spindle is also a subject of research since its first description [14] with data supporting its sleep preservation role as an arousal inhibitor [15]. The importance of understanding the mechanisms underlying KCs, spindles and their possible interaction extends also beyond their role in sleep maintenance, as they have been proposed to be implicated in memory consolidation [16], stroke and spindle-coma [17], schizophrenia [18] and epilepsy [1], [2], [19], [20].

The relationship between KCs and spindles has been described as antagonistic. Administration of benzodiazepines increases spindle appearance and decreases KCs [21]–[23]. In a period of 10 s before transient arousals, the incidence of spontaneous KCs increases while there is a decrease of both isolated sleep spindles and of spindles associated with KCs [24], [25]. Halasz [13] reported a suppression of spindles power for 5–15 s following evoked KCs that were part of a microarousal, thus proposing that these states allow a window of improved sensory inflow at the thalamocortical (TC) circuits while preserving sleep continuity. KC is also seen as the forerunner of delta waves of slow-wave sleep (SWS) and this scheme resembles the reciprocal relationship of sleep spindles and delta waves [3], [26]. Curcio et al [27] showed an increase of sleep spindles throughout the night while the occurrence of spontaneous KC decreased. Other studies support independent roles for spindles and KCs. Following stroke spindles disappear while KCs remain [28]. Church et al [29] found that there is no suppression of evoked KC by spindles, a result confirmed by Crowley et al [30]. In the underlying network level, sleep spindles are paced by TC networks whereas KCs by intracortical networks [31], independently from the thalamus [32] (but see Crunelli et al [33] and Bonjean et al [34]).

Kokkinos and Kostopoulos [35] using time-frequency analysis (TFA) showed that fast spindles which happen to coincide with spontaneous KCs are interrupted, during that interruption a slower oscillation most often appears over the negative peak of the KC and spindles following KCs always had a higher spectral frequency than both interrupted and isolated sporadic fast sleep spindles. These results reveal an interaction on the time level of about a second, nearly the duration of a KC. Possible interactions of evoked KCs and sleep spindles on a longer time frame were reported by Halasz [13] but not confirmed by Bastien et al [36]. Zygierewicz et al [37] described a reduction on spindle power 3.5 s post-stimulus on responses containing evoked KCs, but limited the analysis up to 5 s post-stimulus. A long term depressant effect of spontaneous KCs on spindle generation would suggest that KCs by themselves may tend to disrupt sleep maintenance. The main objective of this study was to assess interactions of spontaneous rather than evoked KCs and spindles on similar time scales of 15 s applying event-related methodology and detailed TFA.

## Materials and Methods

### Ethics Statement

This research has been approved by the University of Patras Committee for Ethics in Research. All participants provided written informed consent to the procedures and their data were anonymously processed.

### Subjects, Procedures and Recording

Seven volunteers (2 males and 5 females, mean age 26.3, range 23 to 33 years) were included in the present study. There was no report of neurological, psychiatric or sleep disorder in their medical history and at the time of study all were in good health and free from any medication. The participants kept a sleep diary for a week, were instructed to refrain from alcohol and caffeine for at least 3 and 1 days prior to the experiment respectively and follow their regular sleep schedule. They had no difficulties in falling or remaining asleep during the night and all were good sleepers. Subjects were instructed to arrive at the laboratory approximately 1 hour prior to their usual bedtime, as calculated on average based on their sleep diaries. Each of them spent the night in an air-conditioned, temperature-controlled, soundproof and dark room. Night sleep recording begun after lights were willingly switched off, and ended with the subjects' spontaneous wake-up in the morning. Whole night recordings included 58 EEG channels, EOG and EMG as well as triggers from a motion-detector over the bed area. All experimental procedures and technical details of the EEG recording have been described elsewhere [35] – that study also includes four subjects of the current work.

### Scoring and Event Selection

**Figure 1:**
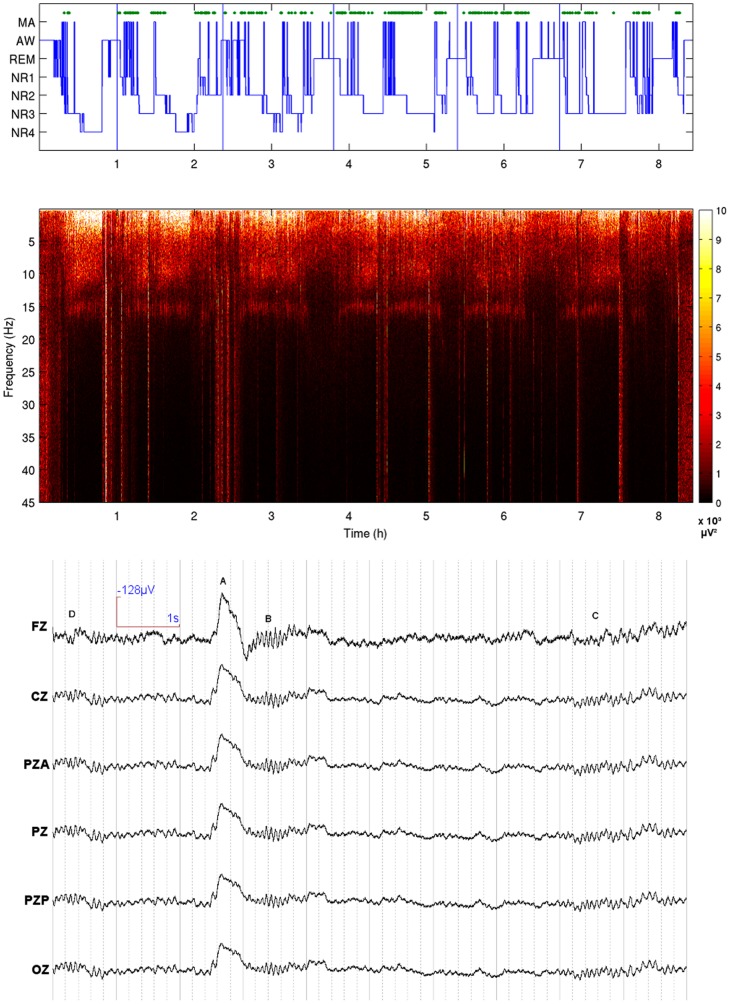
Hypnogram (top) and its respective hypnospectrogram (whole-night time frequency plot of EEG power) (middle) derived from Cz for subject 2. In hypnogram green dots mark the occurrence of KCs selected for the study and vertical lines the definition of a “cycle” used in Figure 2. MA, microarousal, AW, awake, REM, rapid-eye movement sleep, NR1–4, non-REM sleep stages 1–4. Bottom part: Raw EEG of selected midline electrodes. A K-complex (A) from NREM stage II ending with a spindle (B) is seen (group KC_01_). Two individual sporadic spindles are also seen (C, D). D is not included in this study because of its proximity to the KC. Sleep staging for all the subjects is provided as a lasagna plot [52] in supplementary figure.

Sleep staging was performed by visual inspection according to the standard criteria of Rechtshaffen and Kales [38], taking under consideration the propositions of the AASM Visual Scoring Task Force [39] as well as those of the DGSM Task Force [40], and the guidelines of the ASDA Report [41] to identify microarousals. Scoring was further aided by the collation of a hypnospectrogram [42], that is, the whole-night FFT-based time-frequency plot for 0.05–45 Hz with a step frequency of 0.05Hz. Continuous scoring with a step of only 1 s was performed rather than epoch-based scoring in order to obtain a precise match between the derived hypnogram and the hypnospectrogram (Fig. 1).

The K-complex was identified as a >500 ms well-delineated negative sharp wave usually followed by a positive phase that stands out of the EEG background (Fig. 1). In this study, singular (without another K-complex or slow wave activity immediately preceding or following) generalized (distinguishable in the EEG all across the midline electrodes) spontaneously occurring K-complexes from NREM stage II and III were selected. A further classification scheme was adopted for the needs of the analysis, using a 2-digit binary subscript KC_X–X+_ denoting absence (0) or existence (1) of coinciding oscillations. The first digit refers to a spindle interrupted by the K-Complex, and the second refers to a spindle starting during the descending negative and the positive phase of the K-complex (this is similar to Kokkinos and Kostopoulos [35], where a third digit is used as a reference to an intra-KC oscillation). K-complexes immediately preceding microarousals and awakenings during sleep, as well as K-complexes followed by delta waves, were excluded from this study.

The sleep spindle was identified as a >500 ms train of ≈11–16 Hz waves. Two types of sleep spindles were further identified, slow and fast spindles, according to the definitions of Gibbs and Gibbs [4]. Fast spindles (>13Hz) exhibit a symmetric bilateral distribution over centro-parietal areas, while slow spindles (<13 Hz) exhibit a similarly bilateral distribution frontally and are absent or significantly diminished in the centro-parietal and posterior areas. In this study, only fast spindles away (±3 s) from K complexes and other delta activity were included, selected from NREM stage II and III (Fig. 1).

### Analysis

Manual cursor marking offered by Scan software (Neuroscan Inc, Charlotte, NC, USA) was used in order to define events. NREM stage II epochs from the whole-night sleep recording were selected and precise time-markers were placed over the events under study. Two kinds of events were visually marked and used for further analysis: a) the peak of the negative phase of the K-complex, b) the peak of the negative wave near the middle of the individual fast spindle (first and last peak of the spindle were visually identified and marked). The peak was marked over the record of the Cz electrode, where fast spindles are prominent.

Event-related data were further processed by a software toolbox for Matlab (The Mathworks, Natick, MA, USA) developed at the Neurophysiology Unit. Event-related TFA was performed for each selected event within a time-window of 60 s centered (time = 0.00) at the marked event. Spectral estimates for time-frequency bins with time resolution 0.0384 s and frequency range from 0.05 to 20 Hz at a step of 0.05 Hz were obtained using a discrete Fourier transformation. Analysis resulted in averaging of the time-frequency plots for all samples for each category of events. No filter was applied to the processed electrophysiological data.

Statistical significance of patterns in the time-frequency plots was assessed by the method described by Zygierewicz et al [37]. Time-frequency elements with resolution of 0.250 s and 2 Hz were calculated using the corresponding mean spectral values, and the Box-Cox transformation was used to transform the values across events to approximately normal distribution. For each element, the null hypothesis of no change from a baseline period −15 to −5 s prior to the event marker was tested using t-test, assuming unequal variances (Welch t-test). Correction for multiple comparisons was performed by controlling false discovery rate [43] with q = 0.05 so that among all significant time-frequency elements 5% of them are false positives. Relative changes of spectral power were calculated using the ratio of the original (not transformed) mean values of the power spectral density for every time-frequency bin to the average of the values during the baseline period [44]. The logarithm of this ratio was plotted for significant patterns.

## Results

Hypnograms and hypnospectrograms (Fig. 1) revealed that all subjects had normal sleep (Table 1). A total of 1239 K-complexes and 1162 sleep spindles from NREM stages II and III were identified and included in this study. K-complexes were separated into 4 groups: (a) KCs with spindles identified only just after their negative peak (group KC_01_, n = 619), (b) KCs with spindles identified only just before their negative peak (group KC_10_, n = 132), (c) KCs with spindles identified both before and after their negative peak (KC_11_, n = 255) and (d) KCs with no spindle visually identified either before or after them (group KC_00_, n = 233). These groups are compared to the results for fast spindles appearing as sporadic i.e. clearly away from KCs and delta waves, in order to assess effects possibly related to spindle activity alone rather than effects related to KCs.

**Table 1:** Descriptive Summary of Sleep Patterns.

	Subject 1	Subject 2	Subject 3	Subject 4	Subject 5	Subject 6	Subject 7	Average
TSP (min)	392	489	517	298	685	381	470	462 (±124)
TST (min)	382	461	497	268	666	381	442	442 (±123)
SE (%)	97.4%	94.3%	96.1%	90%	97.2%	100%	94%	96% (±3)
WASO (min)	10	28	20	30	19	0	28	19 (±11)
NREM1 (min – %)	13 (3%)	34 (7%)	24 (5%)	21 (8%)	44 (7%)	6 (2%)	59 (13%)	29 (±18) / 6% (±4)
NREM2 (min – %)	100 (26%)	116 (25%)	164 (33%)	109 (40%)	316 (47%)	152 (40%)	161 (37%)	160 (±74) / 35% (±8)
NREM3 (min – %)	130 (34%)	178 (39%)	88 (18%)	13 (5%)	54 (8%)	34 (9%)	49 (11%)	78 (±58) / 18% (±13)
NREM4 (min – %)	46 (12%)	31 (7%)	80 (16%)	69 (26%)	39 (6%)	108 (28%)	75 (17%)	64 (±27) / 16% (±9)
REM (min – %)	77 (20%)	73 (16%)	121 (24%)	40 (15%)	189 (28%)	71 (19%)	47 (11%)	88 (±51) / 19% (±6)
MA (min – %)	17 (4%)	28 (6%)	20 (4%)	17 (6%)	25 (4%)	10 (3%)	51 (12%)	24 (±13) / 6% (±3)
Fast Spindle Average Frequency	14.55 Hz	15.2 Hz	13.6 Hz	13.95 Hz	13.05 Hz	13.3 Hz	14.2 Hz	14 Hz (±0.75)
KCs included	225 (18%)	259 (21%)	195 (16%)	105 (8%)	163 (13%)	164 (13%)	128 (10%)	177 (±54)
Spindles included	178 (15%)	114 (10%)	228 (20%)	132 (11%)	100 (9%)	255 (22%)	155 (13%)	166 (±58)

Sleep patterns for 7 subjects. TSP: Total Sleep Period, TST: Total Sleep Time, SE: Sleep Efficiency, WASO: Wakefulness after sleep onset, NREM1–4, REM, MA: Minutes in each sleep stage and percentage relative to TST, KCs and spindles included in the study and percentage relative to total number of events included.

**Figure 2:**
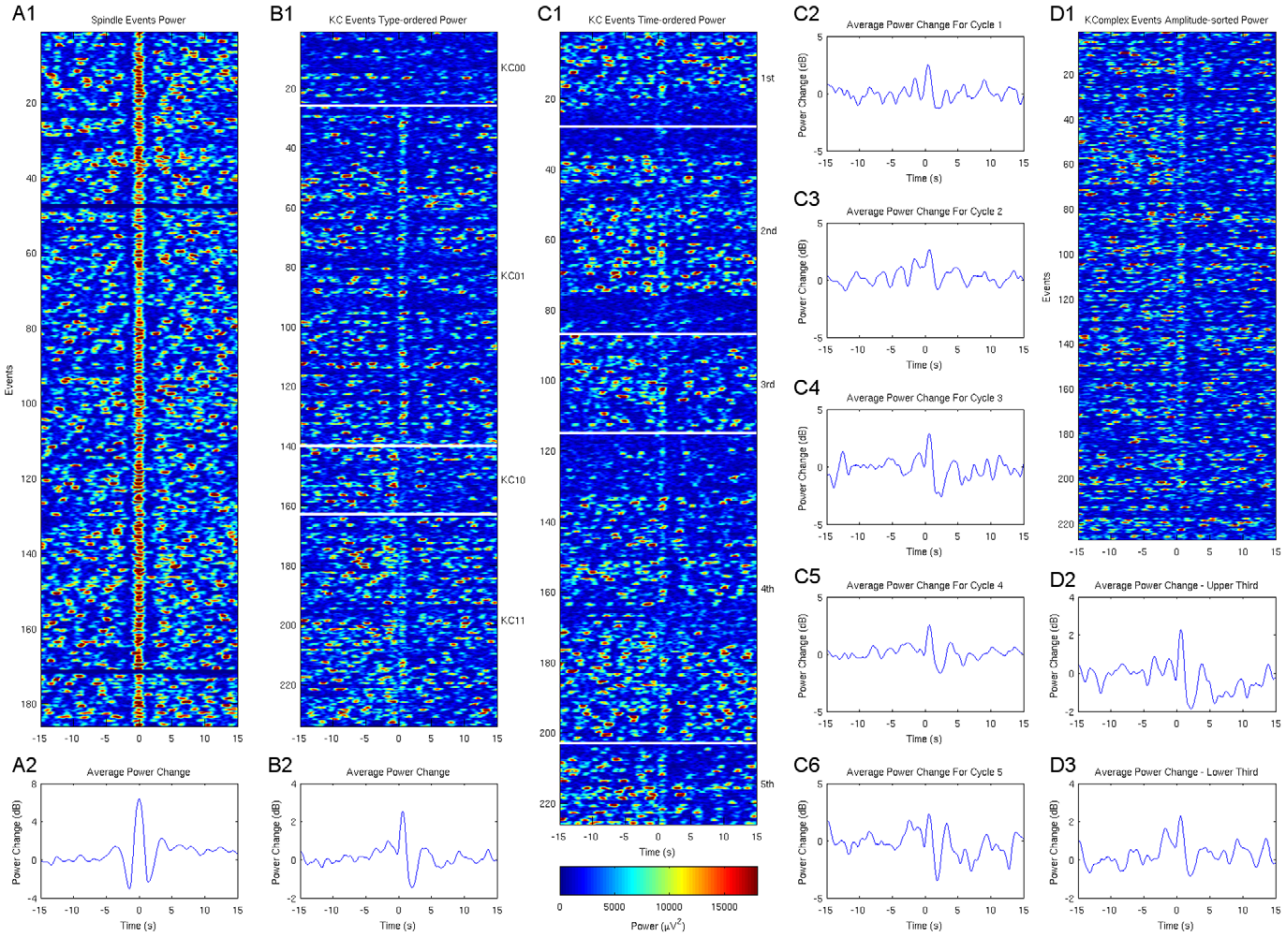
All graphs show Spindle Band Power developing over time: Raster images composed of individual time-frequency plots of EEG power near the frequencies of each subject's individual spindle spectral frequency band, for 15 s before and after each event (sporadic spindles in A and KCs in B–D). Average power change is shown below each raster. **A1–2:** Spindles as reference events (at time zero). In the y-axis spindle event successive number; all averaged in A2. **B1–2:** KCs as reference events, spindle data sorted by KC group (from top to bottom: KC_00_, KC_01_, KC_10_, KC_11_); all averaged in B2. **C1–6:** KCs as reference events, spindle data sorted by KCs time of occurrence during the night and separated in successive sleep cycles; data from cycles 1–5 averaged in C2–C6 respectively. **D1–3:** KCs as reference events, spindles data sorted by the amplitude of KCs negative peak. D2 and D3 average data for the relatively larger and smaller KCs respectively. Relative absence of spindles is prominent 2–3 s after the negative peak (B1,C1,D1) and a relative long-term (10–15 s) reduction in their rate of appearance is shown for the about 80 top amplitude-sorted KCs (D1–3). All images, from subject 1.

Spindles spectral frequency is stable for each subject but varies between subjects [45]. Therefore for every subject, the average power spectral density graph of one-minute EEG segments around all of the markers was used to determine the individual fast spindle frequency band and select a band width of 1.5 Hz encompassing the peak of the PSD. Focusing on these frequency limits, TFA plots of EEG segments around individual reference events (KCs or spindles) were placed on a parallel formation to compose a raster image (Fig. 2). Using individual fast spindles as reference events, a raster image of spindle power distribution around fast spindles was obtained (Fig. 2 A) and compared to distributions obtained for KCs as reference events sorted by KC group, time of occurrence and negative peak amplitude (Fig. 2 B, C, D). These raster images were expected to visualize any patterns of non-random distribution of spindle activity around KCs. In Fig. 2 A, time zero marks the middle of spindles which are presented as a thin red vertical band. An absence of spindles for about 2–3 s before and after the individual sporadic spindles is observed. In Fig. 2 B, C, D time zero marks the KC negative peak. Spindles associated with KCs form a vertical line near zero. The short-term absence of spindles right after this line, about 2–3 s after the KC negative peak, is observed in this case as well. Though less prominent in some, this result was obvious in all 7 subjects. Moreover, in 6 out of 7 subjects (less clear in subject 5), there were clusters of events in which the spindles in a period lasting 10–15 s after the KC were less when compared to a baseline period −15 to −5 s before the KC. However, this long-term relation did not apply to all the events, nor was obvious in all subjects. In one subject, sorting the KCs by the amplitude of the negative peak revealed that this long-term effect was more prominent in the KCs with the highest peak amplitude (Fig. 2 D), but this was not repeated in the other subjects.

**Figure 3:**
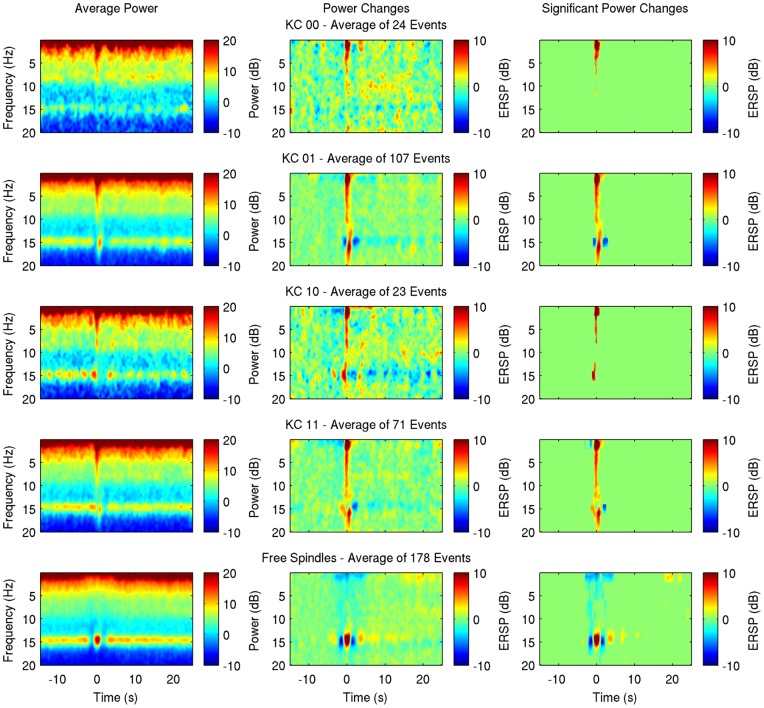
Average spectrogram (left), event-related spectral perturbation (middle) and significant changes (right) for a time period 15 s before and 25 s after the negative peak of KCs sorted by group (KC_00_, KC_01_, KC_10_, KC_11_ in rows 1–4 respectively) and the negative middle peak for sporadic spindles (in 5th row) of subject 1.

**Figure 4:**
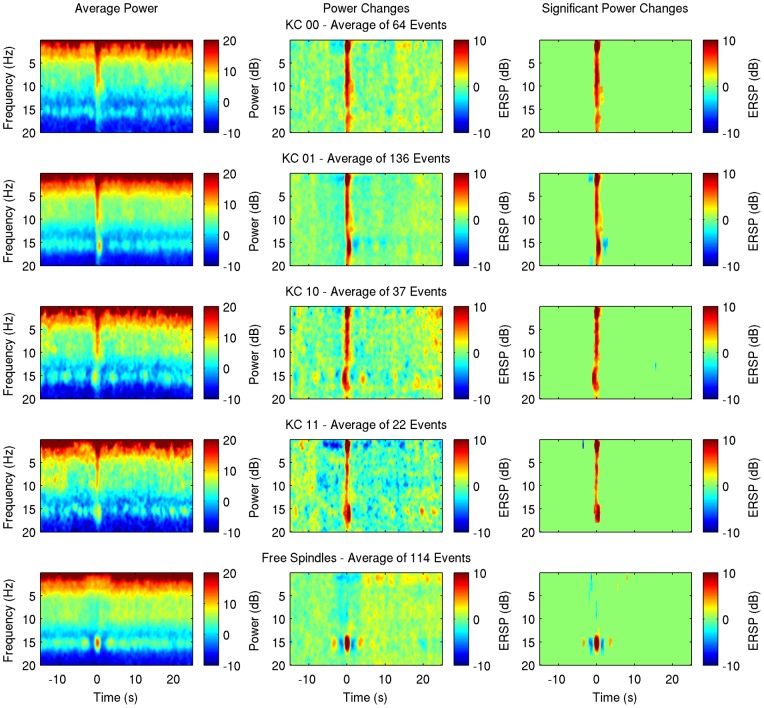
Average spectrogram (left), event-related spectral perturbation (middle) and significant changes (right) as in Fig. 3 but for subject 2.

Following the initial qualitative analysis, the average spectrogram, relative changes and statistically significant time-frequency bins [37] were calculated for every subject and every group (Fig. 3–4 for subjects 1, and 2, supplementary figures for subjects 3–7). The baseline period is defined as −15 to −5 s prior to the event.

As Kokkinos and Kostopoulos [35] described, the spectral effects of the KC itself consist of a major increase of power on the lower delta band which extends to higher frequencies with prominent increase near 5–10 Hz in all classes. The interruption of spindles during the KC and the faster spindle after the KC negative peak described by Kokkinos and Kostopoulos [35] are obvious in the TFA plots of KC_11_ group, comprised of KCs with spindles appearing both right before and after the negative peak.

Individual sporadic spindles analysis revealed a pattern of increase in spindle power followed by a decrease before the spindle and a symmetrical decrease followed by an increase after the spindle in all subjects (in subject 4 without reaching statistical significance), therefore suggesting a refractory period lasting 1.5–2 s. The pattern of a short-term decrease in spindle power after an initial increase is seen in KC_01_ group as well, nearly 2 to 3 seconds after the KC in all subjects, though it reaches statistical significance in 4 out of 7 subjects (Subjects 1, 2, 3, 6). In these subjects a closer look reveals repeated decreases every 3–4 s lasting for a period of about 15 s, a result that reaches significance in subject 6. In subject 1, where a significant decrease of spindle power is shown just prior to the KC (of course this group is selected to not have spindles prior to the KC), the same 3 s interval appears. In KC_10_ group the expected increase of spindle power prior to the KC is obvious, and though the number of events in this group is smaller, in subjects 1, 2, 3, 4 and 7 there is a suggestion of decrease of spindle power nearly 3 s before the KC. A pattern of rhythmic decreases also appears but without reaching significance. In KC_11_ group, the short-term decrease on spindle power 2–3 s after the KC is statistically significant in one subject (subject 1) only, and the pattern of rhythmic decreases is seen in subjects 1, 3, 6, 7.

In group KC_00_, there is no long term change on spindle power after the KC. During the time around a KC (+− 1 s), 2 subjects (2 and 5) show on average an increased power in the sigma band, though spindles could not be detected visually on the raw EEG. In 3 subjects (2, 4, 5) an increase in higher frequencies (> 15Hz) is also observed during the KC.

**Figure 5:**
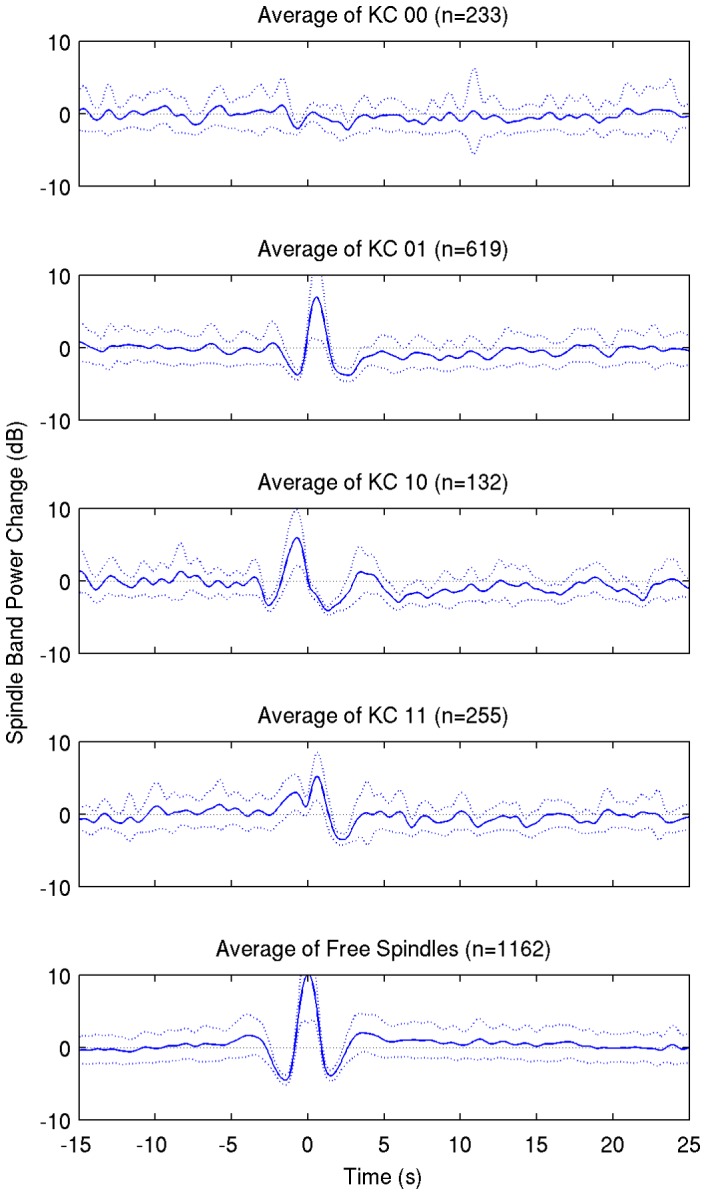
Grand average of spindle power changes (dark blue line) ± SD on all KC groups (rows 1–4) and individual spindles (5th row) for all subjects. The average change is calculated over the individual spindle frequency band for every subject.

No significant long-term decrease of spindle power was detected in any of the subjects, so in order to facilitate visualization, the average band power for each subject's individual frequency band was calculated and changes of the grand average power relative to baseline are presented for every group (Fig. 5). The short-term effect is seen on spontaneous KCs associated with spindles (KC_01_, KC_10_, KC_11_) and on free fast spindles as well, but not on KCs not accompanied by spindles (KC_00_).

In group KC_01_ where the number of events is larger and the trace of power change is smoother, there is a very small decrease of −1 dB in spindle power relative to baseline lasting more than 15 s. The trace reaches zero (no change from baseline) nearly 20 s after the KC peak. As shown for subject 1, a cluster of events including the larger KCs exhibits a long-term reduction (Fig. 2, D2 vs D3). In other subjects, similar clusters appear, but do not seem correlated to either KC negative peak amplitude or time of occurrence. These clusters may account for the small long-term reduction maintained in the grand average.

Also note that in all KC groups, the TFA maps do not show any change in the time frame −5 to 0 s before the KC relative to baseline that could support any factor on the frequency range studied (0–20Hz) able to predict the appearance of a K-complex.

## Discussion

We have examined a total of 2401 EEG events (including both epochs with spontaneous KCs and epochs with only free fast spindles) taken from 7 subjects using TFA. The analysis included examination of the pattern of spindle power distribution around KCs, clustering of KCs based on spindle appearance within a second of the negative peak and detailed TFA for 40 s focusing on 0–20 Hz with respective statistical analysis, and finally, comparison to individual sporadic fast spindles.

The pattern of spindle distribution around KCs (Fig. 2) reveals a short-term reduction in power 2–3 s after the KC negative peak and clusters of events where a long-term reduction (10–15 s) is visible. However, as shown on Figure 5, on average of all events the long-term effect is very small (in group KC_01_) or non-existent (in all other groups).

Time-frequency average results (Fig. 3–4) reveal a short-term event related desynchronization (ERD), 2–3 s after the negative peak of the KC. This is obvious and significant on groups of KCs accompanied by post-KC spindles (KC_01_ and KC_11_) and is similar to a same ERD that follows individual sporadic sleep spindles. This result is also seen on evoked KCs in 2/3 subjects of Zygierewicz et al [37] after an event related synchronization at spindle frequency range, however the authors do not present data of evoked KCs not followed by spindles. It seems that this is not an effect of the KC per se. Instead these data suggest a refractory period of spindles independently of KCs and in conjunction with our data on sporadic fast spindles (Fig. 2A and Fig. 5) this finding is rather related to a rhythm of about 0.25–0.3 Hz underlying sleep spindle occurrence.

The refractoriness of spindles for 3–20 s has been shown in vitro. More than one local spindle oscillations can be independently generated in thalamic slices and their local propagation and the stoppage of spindle propagation at the point of collision both indicate the presence of a refractory period for spindle wave generation and propagation [46]. This refractory period has been attributed to an afterdepolarization of thalamic neurons after their intense hyperpolarization/bursting during spindles. McCormick and Bal [46] more specifically suggest that the spindle refractory period is the time required for the h-current to return to a level that allows another spindle wave to occur. However at the human EEG level additional factors may determine the spindles refractory period, like the degree of global synchronization needed for spindles to be detected on EEG, depending on physical factors related to spindles’ current sources orientation and volume conduction [47]. Furthermore there is ample evidence for a role of corticothalamic input in both the initiation and the termination of spindle oscillations [34]. This cortical input may conceivably be random in light NREM sleep or be periodic following a slow cortical oscillation [48] in the case of spindles arising during slow wave (3d stage of NREM) sleep. Experimental evidence suggests that the spindles instigating cortical excitation of reticular thalamic neurons is most often elicited during the transition from cortical “down” to cortical “up” state. This may apply to our observations which are made on spontaneous isolated KCs, since human studies have shown that KCs may be isolated down states (Cash et al., 2009). Finally spindles can be induced or modulated locally, but also remotely (hippocampal-frontal dialogue), and vary in density according to sleep pressure and many other factors. A periodic emergence of spindles appears therefore to be the result of an interaction between several cortical and subcortical mechanisms, whose balance may vary in brain space and in sleep time.

Spindle periodicity has been shown earlier: Evans and Richardson [49] have reported a periodicity of 3–5 s by measuring intervals between spindle bursts, which is compatible to our results of the short-term ERD seen in the TFA maps of KCs, especially KC_01_ group, and in the pattern shown on individual sporadic spindles. Achermann and Borbely [50] have detected this rhythm with FFT analysis. Zygierewicz et al [37] also report the same interval between the ERDs before and after the evoked KC.

Regarding a possible long-term interaction of spontaneous KCs with sleep spindles, extending to 10–15 s, our data suggest a very small effect detected on group KC_01_. Compared to the effect of evoked microarousals on sleep spindles reported by Halasz [13], there is no significant similar effect of spontaneous KCs on spindles. Halasz does report a pronounced long-term depression on spindle power of evoked microarousals, including responses of single KC not associated with spindles, but, interestingly, only a slight depression in their KS group, which the author defines as “K-complex followed by or intermingled with 13–14 cps sigma spindle”. Our results for spontaneous KC_01_, KC_10_ and KC_11_ are similar to this long-term slight depression of spindles power for evoked KS group. However, the results of our spontaneous KC_00_ are different from their evoked single K-complex. As for the short-term effect, note that in the figures provided by Halasz, an ERD can be also seen almost 3 s post-stimulus.

Bastien et al [36] have also examined spindle power before and after evoked KCs. In their data they did not detect differences between 4 seconds pre-stimulus and either short-term, 1.25–5.25 s, or long-term, 5.26–9.25 s post-stimulus effects. The differences on the methodology of the EEG analysis of these studies do not allow solid conclusions on the possible long-term effects of evoked KCs on sleep spindles and a direct comparison to our data on spontaneous KCs. These differences include our individual spindle frequency approach i.e. the use of a different frequency band as specifically measured for each subject. For example, the 14Hz used by Halasz [13] are not included in the bands we used for 2 of our subjects (subjects 2 and 5) and the 12–14Hz used by Bastien et al [36] would not include the spindles of one subject (subject 2). Time resolution is also an important factor. Bastien et al [36] used a 4 s segment FFT that would probably not detect our short-term ERD and Halasz [13] used 1-s FFT, compared to our 0.25 s bins for statistical analysis and of course finer initial spectrograms.

Clustering of spontaneous K complexes based on the incidence of spindles in close time proximity to KCs, may also be a factor to understanding their interactions. Our KC groups (see also Kokkinos and Kostopoulos [35]) are similar to the classification of Ehrhart et al [25] who separate KCs to those without contiguously occurring spindles and KCs with sleep spindles occurring just prior, during and just after the KC, in order to assert their relation to transient arousals.

In conclusion, single spontaneous KCs that do not lead to microarousals interact with spindles only on a short time scale of about a second [35] but we could not detect long-term spindle power reduction, extending to 10–15 s, as pronounced as in the case of evoked KCs [13]. Evoked KCs that are accompanied by spindles (KS group of Halasz [13]) also do not display the long-term sustained inhibition of spindles. Our results after clustering of spontaneous KCs according to their amplitude or their short term relationship to spindles, also suggest that any long term effects of evoked KCs to spindles is probably not related to KCs per se but to the stimulus and/or the other components of the longer phasic event it usually elicits. The importance of the distinction made in this study lies with the role of spontaneous KCs in sleep maintenance, as well as with the demonstrated involvement of spindles in several cognitive functions and their increasing association to several neuropsychiatric disorders.

Finally, the time-frequency maps do not show any change before the KC (time frame −5 to 0 s) that could support any factor on the frequency range studied (0–20Hz) able to predict the appearance of a K-complex, as is reported for higher (>20Hz) frequencies and evoked KCs [51].

## Supporting Information

Figure S1 Hypnograms for all 7 subjects. Each row represents one subject and sleep stages are color-coded. Microarousals are not shown.
(TIF)

Figure S2 Average spectrogram (left), event-related spectral perturbation (middle) and significant changes (right) for subject 3.
(TIF)

Figure S3 Average spectrogram (left), event-related spectral perturbation (middle) and significant changes (right) for subject 4.
(TIF)

Figure S4 Average spectrogram (left), event-related spectral perturbation (middle) and significant changes (right) for subject 5.
(TIF)

Figure S5 Average spectrogram (left), event-related spectral perturbation (middle) and significant changes (right) for subject 6.
(TIF)

Figure S6 Average spectrogram (left), event-related spectral perturbation (middle) and significant changes (right) for subject 7.
(TIF)

## References

[1] Halász P (2005) K-complex, a reactive EEG graphoelement of NREM sleep: an old chap in a new garment. Sleep Med Rev 9: 391–412 doi:10.1016/j.smrv.2005.04.003. 16122950 10.1016/j.smrv.2005.04.003

[2] Colrain IM (2005) The K-complex: a 7-decade history. Sleep 28: 255–273. 16171251 10.1093/sleep/28.2.255

[3] De Gennaro L, Ferrara M (2003) Sleep spindles: an overview. Sleep Med Rev 7: 423–440. 14573378 10.1053/smrv.2002.0252

[4] Gibbs FA, Gibbs EL (1950) Atlas of electroencephalography Vol. 1, Methodology and controls. Reading, MA: Addison-Wesley.

[5] Anderer P, Klösch G, Gruber G, Trenker E, Pascual-Marqui RD, et al (2001) Low-resolution brain electromagnetic tomography revealed simultaneously active frontal and parietal sleep spindle sources in the human cortex. Neuroscience 103: 581–592. 11274780 10.1016/s0306-4522(01)00028-8

[6] Schabus M, Dang-Vu TT, Albouy G, Balteau E, Boly M, et al (2007) Hemodynamic cerebral correlates of sleep spindles during human non-rapid eye movement sleep. Proc Natl Acad Sci USA 104: 13164–13169 doi:10.1073/pnas.0703084104. 17670944 10.1073/pnas.0703084104 PMC1941810

[7] Loomis AL, Harvey EN, Hobart GA (1938) Distribution of disturbance-patterns in the human electroencephalogram, with special reference to sleep. Journal of Neurophysiology 1: 413–430.

[8] Hornyak M, Cejnar M, Elam M, Matousek M, Wallin BG (1991) Sympathetic muscle nerve activity during sleep in man. Brain 114 ( Pt 3): 1281–1295. 10.1093/brain/114.3.1281 2065250

[9] Okada H, Iwase S, Mano T, Sugiyama Y, Watanabe T (1991) Changes in muscle sympathetic nerve activity during sleep in humans. Neurology 41: 1961–1966. 1745356 10.1212/wnl.41.12.1961

[10] Roth M, Shaw J, Green J (1956) The form voltage distribution and physiological significance of the K-complex. Electroencephalogr Clin Neurophysiol 8: 385–402. 13330651 10.1016/0013-4694(56)90004-9

[11] Takeuchi S, Iwase S, Mano T, Okada H, Sugiyama Y, et al (1994) Sleep-related changes in human muscle and skin sympathetic nerve activities. J Auton Nerv Syst 47: 121–129. 8188978 10.1016/0165-1838(94)90073-6

[12] Tank J, Diedrich A, Hale N, Niaz FE, Furlan R, et al (2003) Relationship between blood pressure, sleep K-complexes, and muscle sympathetic nerve activity in humans. Am J Physiol Regul Integr Comp Physiol 285: R208–214 doi:10.1152/ajpregu.00013.2003. 12793998 10.1152/ajpregu.00013.2003

[13] Halász P (1993) Arousals without awakening – dynamic aspect of sleep. Physiol Behav 54: 795–802. 8248359 10.1016/0031-9384(93)90094-v

[14] Loomis AL, Harvey EN, Hobart GA (1936) Electrical potentials of the human brain. J Exp Psychol 19: 249–279.

[15] Yamadori A (1971) Role of the spindles in the onset of sleep. Kobe J Med Sci 17: 97–111. 5147784

[16] Fogel SM, Smith CT (2011) The function of the sleep spindle: a physiological index of intelligence and a mechanism for sleep-dependent memory consolidation. Neurosci Biobehav Rev 35: 1154–1165 doi:10.1016/j.neubiorev.2010.12.003. 21167865 10.1016/j.neubiorev.2010.12.003

[17] Urakami Y (2012) Relationship between, sleep spindles and clinical recovery in patients with traumatic brain injury: a simultaneous EEG and MEG study. Clin EEG Neurosci 43: 39–47. 22423550 10.1177/1550059411428718

[18] Ferrarelli F, Huber R, Peterson MJ, Massimini M, Murphy M, et al (2007) Reduced sleep spindle activity in schizophrenia patients. Am J Psychiatry 164: 483–492 doi:10.1176/appi.ajp.164.3.483. 17329474 10.1176/ajp.2007.164.3.483

[19] Kostopoulos GK (2000) Spike-and-wave discharges of absence seizures as a transformation of sleep spindles: the continuing development of a hypothesis. Clin Neurophysiol 111 Suppl 2S27–38. 10996552 10.1016/s1388-2457(00)00399-0

[20] Si Y, Liu L, Li Q, Mu J, Tian L-Y, et al (2010) Features of the K-complex waves in refractory nocturnal frontal lobe epilepsy. Epilepsy Res 92: 219–225 doi:10.1016/j.eplepsyres.2010.10.002. 21071178 10.1016/j.eplepsyres.2010.10.002

[21] Gaillard JM, Tissot R (1975) EEG sleep studies of insomniacs under flunitrazepam treatment. Int Pharmacopsychiatry 10: 199–207. 2563 10.1159/000468195

[22] Johnson LC, Hanson K, Bickford RG (1976) Effect of flurazepam on sleep spindles and K-complexes. Electroencephalogr Clin Neurophysiol 40: 67–77. 55349 10.1016/0013-4694(76)90180-2

[23] Kubicki S, Haag-Wüsthof C, Röhmel J, Herrmann WM, Scheuler W (1987) Effect of lormetazepam, triazolam and flunitrazepam on rapid eye movements, K-complexes and sleep spindles in normal probands. EEG EMG Z Elektroenzephalogr Elektromyogr Verwandte Geb 18: 61–67. 3111828

[24] Naitoh P, Antony-Baas V, Muzet A, Ehrhart J (1982) Dynamic relation of sleep spindles and K-complexes to spontaneous phasic arousal in sleeping human subjects. Sleep 5: 58–72. 7071452 10.1093/sleep/5.1.58

[25] Ehrhart J, Ehrhart M, Muzet A, Schieber JP, Naitoh P (1981) K-complexes and sleep spindles before transient activation during sleep. Sleep 4: 400–407. 7313393

[26] De Gennaro L, Ferrara M, Bertini M (2000) The spontaneous K-complex during stage 2 sleep: is it the “forerunner” of delta waves? Neurosci Lett 291: 41–43. 10962149 10.1016/s0304-3940(00)01366-5

[27] Curcio G, Ferrara M, Pellicciari MC, Cristiani R, De Gennaro L (2003) Effect of total sleep deprivation on the landmarks of stage 2 sleep. Clin Neurophysiol 114: 2279–2285. 14652087 10.1016/s1388-2457(03)00276-1

[28] Rossetti AO, Maeder-Ingvar M, Reichhart MD, Despland P-A, Bogousslavsky J (2005) Transitory sleep spindles impairment in deep cerebral venous thrombosis. Neurophysiol Clin 35: 19–23 doi:10.1016/j.neucli.2004.12.003. 15808964 10.1016/j.neucli.2004.12.003

[29] Church MW, Johnson LC, Seales DM (1978) Evoked K-complexes and cardiovascular responses to spindle-synchronous and spindle-asynchronous stimulus clicks during NREM sleep. Electroencephalogr Clin Neurophysiol 45: 443–453. 81747 10.1016/0013-4694(78)90289-4

[30] Crowley K, Trinder J, Colrain IM (2004) Evoked K-complex generation: the impact of sleep spindles and age. Clin Neurophysiol 115: 471–476. 14744590 10.1016/j.clinph.2003.10.014

[31] Cash SS, Halgren E, Dehghani N, Rossetti AO, Thesen T, et al (2009) The human K-complex represents an isolated cortical down-state. Science 324: 1084–1087 doi:10.1126/science.1169626. 19461004 10.1126/science.1169626 PMC3715654

[32] Amzica F, Steriade M (2002) The functional significance of K-complexes. Sleep Med Rev 6: 139–149. 12531149 10.1053/smrv.2001.0181

[33] Crunelli V, Cope DW, Hughes SW (2006) Thalamic T-type Ca2+ channels and NREM sleep. Cell Calcium 40: 175–190 doi:10.1016/j.ceca.2006.04.022. 16777223 10.1016/j.ceca.2006.04.022 PMC3018590

[34] Bonjean M, Baker T, Lemieux M, Timofeev I, Sejnowski T, et al (2011) Corticothalamic feedback controls sleep spindle duration in vivo. J Neurosci 31: 9124–9134 doi:10.1523/JNEUROSCI.0077-11.2011. 21697364 10.1523/JNEUROSCI.0077-11.2011 PMC3131502

[35] Kokkinos V, Kostopoulos GK (2011) Human non-rapid eye movement stage II sleep spindles are blocked upon spontaneous K-complex coincidence and resume as higher frequency spindles afterwards. J Sleep Res 20: 57–72 doi:10.1111/j.1365-2869.2010.00830.x. 20477951 10.1111/j.1365-2869.2010.00830.x

[36] Bastien CH, Ladouceur C, Campbell KB (2000) EEG characteristics prior to and following the evoked K-Complex. Can J Exp Psychol 54: 255–265. 11195716 10.1037/h0087345

[37] Zygierewicz J, Malinowska U, Suffczyński P, Piotrowski T, Durka PJ (2009) Event-related desynchronization and synchronization in evoked K-complexes. Acta Neurobiol Exp (Wars) 69: 254–261. 19593338 10.55782/ane-2009-1749

[38] Rechtschaffen A, Kales A (1968) A manual of standardized terminology, techniques and scoring system for sleep stages of human subjects. Bethesda, Md.: U.S. Dept. of Health, Education, and Welfare, Public Health Services-National Institutes of Health, National Institute of Neurological Diseases and Blindness, Neurological Information Network.

[39] Silber MH, Ancoli-Israel S, Bonnet MH, Chokroverty S, Grigg-Damberger MM, et al (2007) The visual scoring of sleep in adults. J Clin Sleep Med 3: 121–131. 17557422

[40] Rodenbeck A, Binder R, Geisler P, Danker-Hopfe H, Lund R, et al (2006) A Review of Sleep EEG Patterns. Part I: A Compilation of Amended Rules for Their Visual Recognition according to Rechtschaffen and Kales. Somnologie 10: 159–175 doi:10.1111/j.1439-054X.2006.00101.x.

[41] Bonnet M, Carley D, Carskadon M, Easton P, Guilleminault C, et al (1992) EEG arousals: Scoring rules and examples. A preliminary report from the Sleep Disorders Atlas Task Force of the American Sleep Disorder Association. Sleep 15: 173–184. 11032543

[42] Kokkinos V, Koupparis A, Stavrinou ML, Kostopoulos GK (2009) The hypnospectrogram: An EEG power spectrum based means to concurrently overview the macroscopic and microscopic architecture of human sleep. Journal of Neuroscience Methods 185: 29–38 doi:10.1016/j.jneumeth.2009.09.002. 19747945 10.1016/j.jneumeth.2009.09.002

[43] Durka PJ, Zygierewicz J, Klekowicz H, Ginter J, Blinowska KJ (2004) On the Statistical Significance of Event-Related EEG Desynchronization and Synchronization in the Time-Frequency Plane. IEEE Transactions on Biomedical Engineering 51: 1167–1175 doi:10.1109/TBME.2004.827341. 15248533 10.1109/TBME.2004.827341

[44] Pfurtscheller G, Lopes da Silva FH (1999) Event-related EEG/MEG synchronization and desynchronization: basic principles. Clinical Neurophysiology 110: 1842–1857 doi:10.1016/S1388-2457(99)00141-8. 10576479 10.1016/s1388-2457(99)00141-8

[45] Werth E, Achermann P, Dijk D-J, Borbély AA (1997) Spindle frequency activity in the sleep EEG: individual differences and topographical distribution. Electroencephalography and Clinical Neurophysiology 103: 535–542 doi:10.1016/S0013-4694(97)00070-9. 9402884 10.1016/s0013-4694(97)00070-9

[46] McCormick DA, Bal T (1997) Sleep and arousal: thalamocortical mechanisms. Annu Rev Neurosci 20: 185–215 doi:10.1146/annurev.neuro.20.1.185. 9056712 10.1146/annurev.neuro.20.1.185

[47] Bonjean M, Baker T, Bazhenov M, Cash S, Halgren E, et al (2012) Interactions between core and matrix thalamocortical projections in human sleep spindle synchronization. J Neurosci 32: 5250–5263 doi:10.1523/JNEUROSCI.6141-11.2012. 22496571 10.1523/JNEUROSCI.6141-11.2012 PMC3342310

[48] Steriade M (2006) Grouping of brain rhythms in corticothalamic systems. Neuroscience 137: 1087–1106 doi:10.1016/j.neuroscience.2005.10.029. 16343791 10.1016/j.neuroscience.2005.10.029

[49] Evans BM, Richardson NE (1995) Demonstration of a 3–5s periodicity between the spindle bursts in NREM sleep in man. Journal of Sleep Research 4: 196–197 doi:10.1111/j.1365-2869.1995.tb00169.x.

[50] Achermann P, Borbély A (1997) Low-frequency (<1Hz) oscillations in the human sleep electroencephalogram. Neuroscience 81: 213–222 doi:10.1016/S0306-4522(97)00186-3. 9300413 10.1016/s0306-4522(97)00186-3

[51] Pál I, Simon G, Halász P (1985) K-Complex formation as a function of the ongoing EEG activity. In: Koella W, Rüther E, Schulz H, editors. Sleep ’84. Stuttgart: Gustav Fischer. 232–234.

[52] Swihart BJ, Caffo B, James BD, Strand M, Schwartz BS, et al (2010) Lasagna Plots. Epidemiology 21: 621–625 doi:10.1097/EDE.0b013e3181e5b06a. 20699681 10.1097/EDE.0b013e3181e5b06a PMC2937254

