# Supplementary figures and images for: Spindle Power Is Not Affected after Spontaneous K-Complexes during Human NREM Sleep
Source: PLoS One. 2013 Jan 10;8(1):e54343. doi: 10.1371/journal.pone.0054343 (PMC3542283; doi:10.1371/journal.pone.0054343)

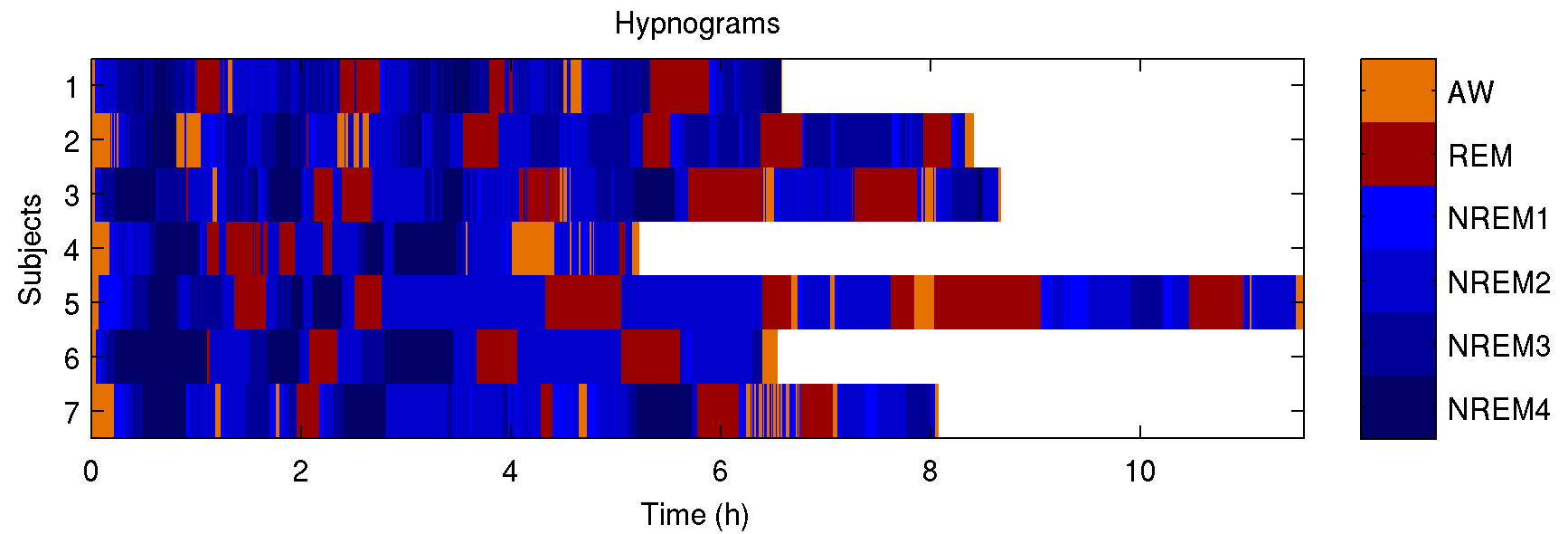

Supplement: Figure S1 — Hypnograms for all 7 subjects. Each row represents one subject and sleep stages are color-coded. Microarousals are not shown. (TIF) [file pone.0054343.s001.tif]

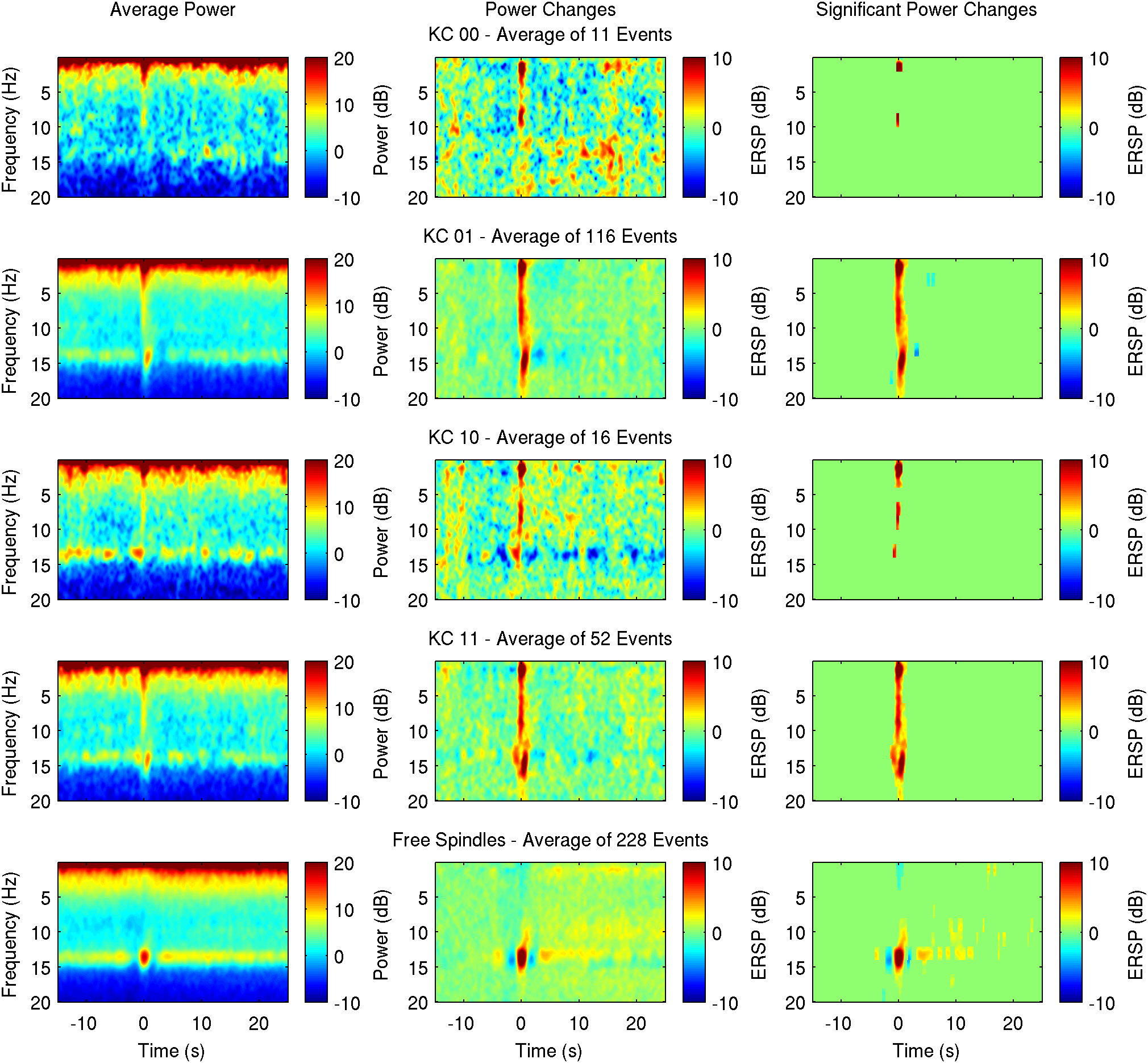

Supplement: Figure S2 — Average spectrogram (left), event-related spectral perturbation (middle) and significant changes (right) for subject 3. (TIF) [file pone.0054343.s002.tif]

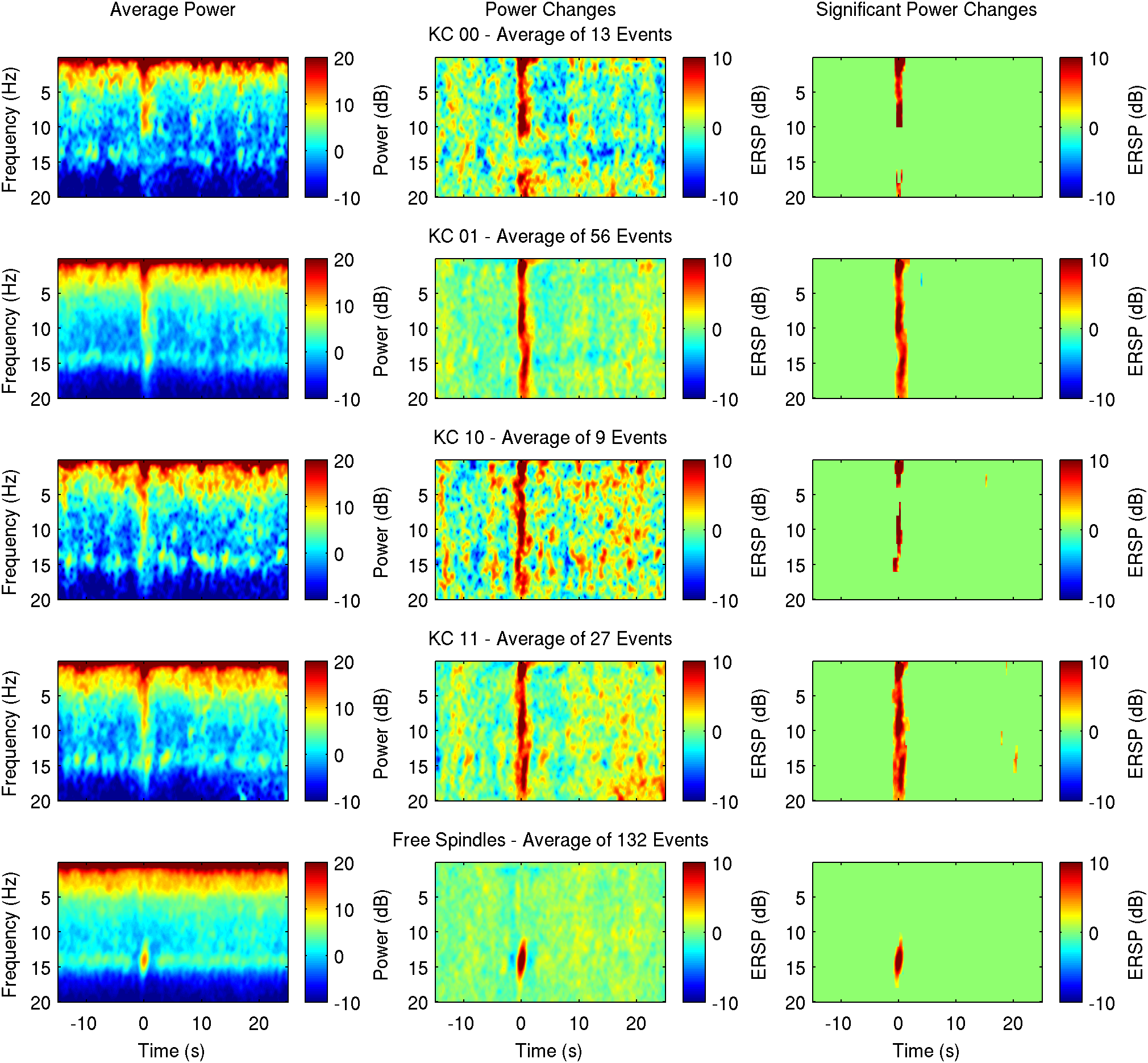

Supplement: Figure S3 — Average spectrogram (left), event-related spectral perturbation (middle) and significant changes (right) for subject 4. (TIF) [file pone.0054343.s003.tif]

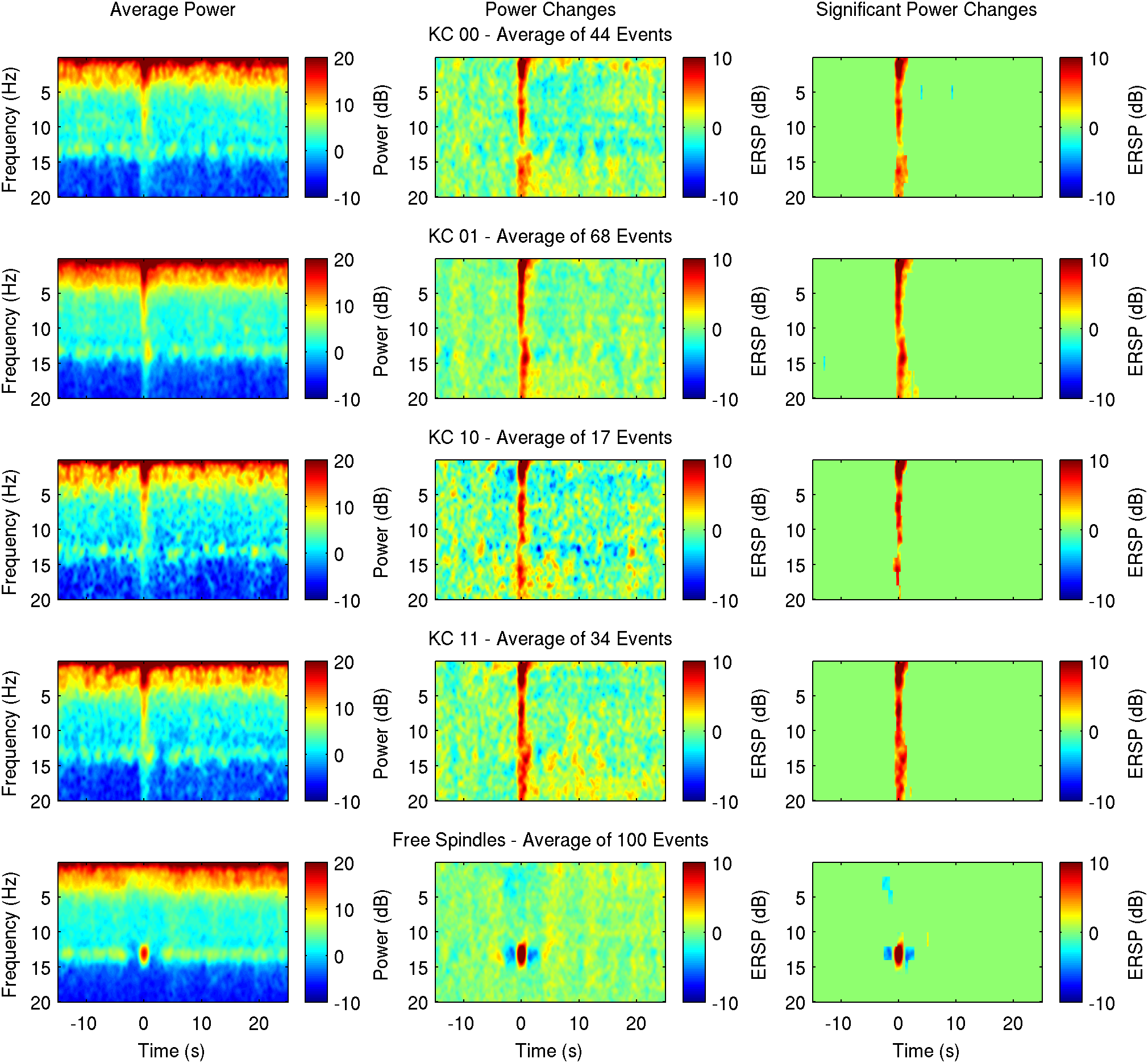

Supplement: Figure S4 — Average spectrogram (left), event-related spectral perturbation (middle) and significant changes (right) for subject 5. (TIF) [file pone.0054343.s004.tif]

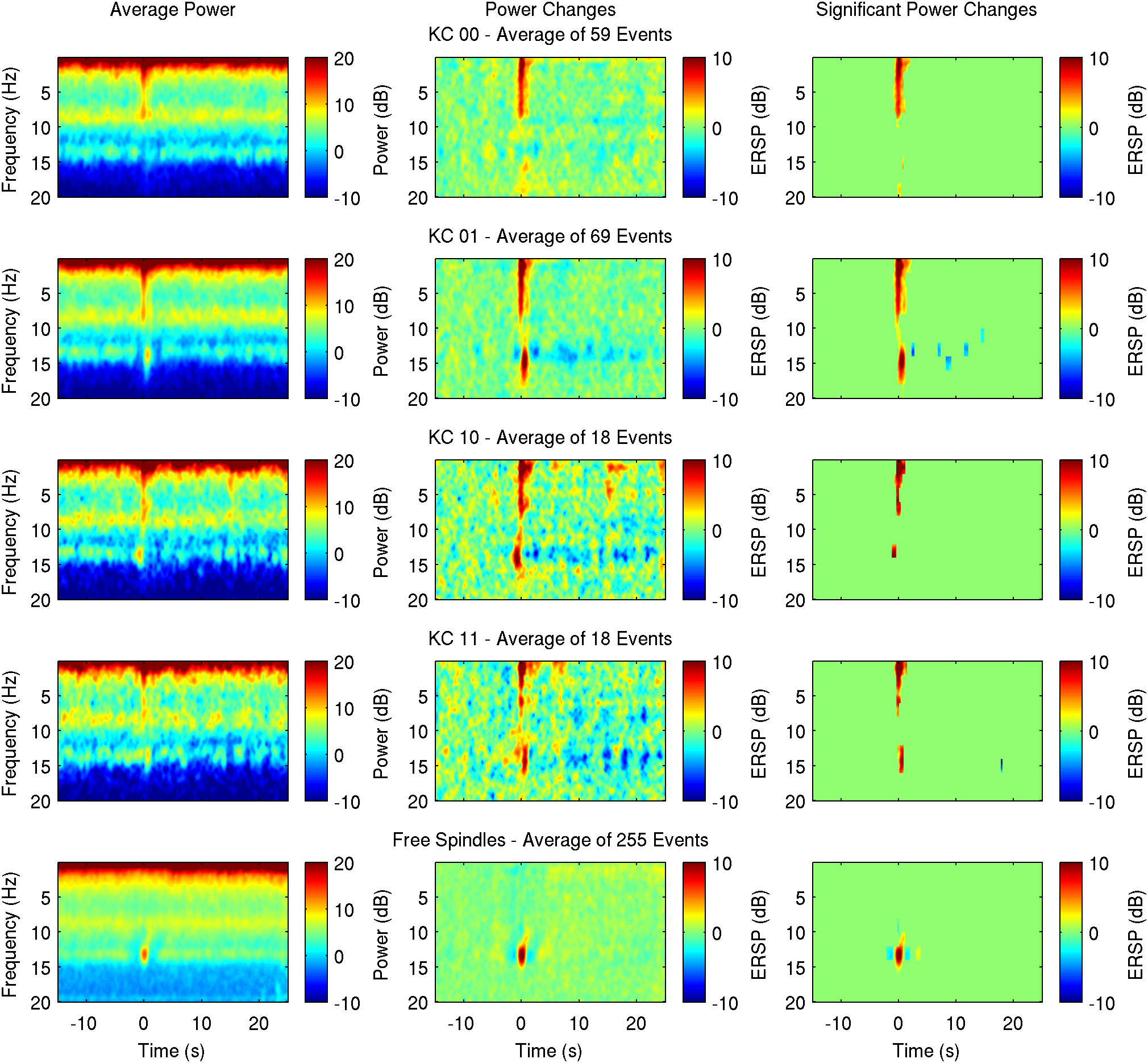

Supplement: Figure S5 — Average spectrogram (left), event-related spectral perturbation (middle) and significant changes (right) for subject 6. (TIF) [file pone.0054343.s005.tif]

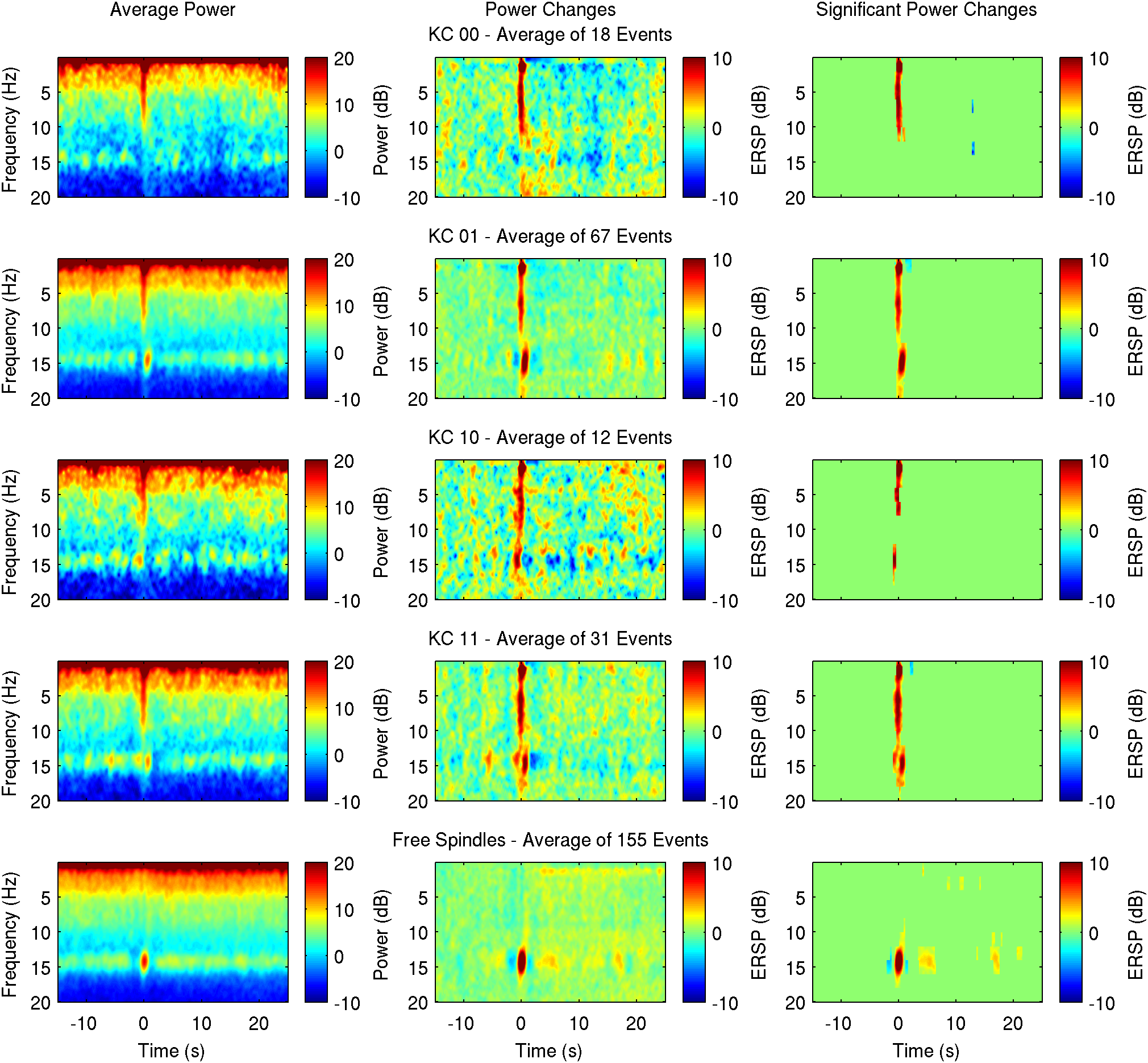

Supplement: Figure S6 — Average spectrogram (left), event-related spectral perturbation (middle) and significant changes (right) for subject 7. (TIF) [file pone.0054343.s006.tif]
